# Impact of Cochlear Trauma Degree and Localization on Intracochlear Electrocochleographic Recordings

**DOI:** 10.3390/audiolres15030074

**Published:** 2025-06-19

**Authors:** David Bächinger, Merlin Schär, Ahmet Kunut, Rahel Bertschinger, Ivo Dobrev, Leanne Sijgers, Andreas H. Eckhard, Adrian Dalbert

**Affiliations:** 1 Department of Otorhinolaryngology, Head and Neck Surgery, University Hospital Zurich, 8091 Zurich, Switzerland; 2 Faculty of Medicine, University of Zurich, 8032 Zurich, Switzerland; 3 Otopathology Laboratory, Department of Otolaryngology, Massachusetts Eye and Ear, Boston, MA 02114, USA; 4 Department of Otolaryngology, Head and Neck Surgery, Harvard Medical School, Boston, MA 02115, USA

**Keywords:** electrocochleography, cochlear implant, cochlear trauma, hearing preservation, intracochlear recordings, mouse model

## Abstract

Background/Objectives: Electrocochleography (ECochG) is a promising tool to monitor preservation of cochlear structures and function during cochlear implant (CI) surgery. However, the interpretation of ECochG signal changes during insertion of the CI electrode array remains controversial. This study investigates the influence of the degree and localization of cochlear trauma on ECochG signal changes using a mouse model. Methods: C57BL/6J-Crl1 mice underwent intracochlear ECochG recordings during the insertion of a platinum–iridium electrode. Results: In case of grade 1 and 2 cochlear trauma, as determined by post-mortem histological analysis, we found that a reduction in intracochlear cochlear microphonic (CM) amplitude correlates more significantly with the location of the trauma than with its severity. The more basally a trauma is located, the larger the CM amplitude drop. Furthermore, the results revealed that grade 1 or 2 trauma was detectable through ECochG before more severe trauma developed. Conclusions: These findings suggest that intracochlear ECochG can serve as a reliable intraoperative tool for detecting early and possibly reversible cochlear trauma, preventing more severe damage and aiding hearing preservation. The results emphasize the need for a nuanced interpretation of CM signal drops, considering trauma location and cochlear structure integrity at the site of trauma and apical to it.

## 1. Introduction

As cochlear implantation has become a widespread option for hearing rehabilitation not only in deaf patients but also in patients with lesser degrees of sensorineural hearing loss, preserving still-intact cochlear structures and function has become one of the primary objectives during cochlear implant (CI) surgery. Preservation of residual hearing not only allows for improved speech perception with electrical stimulation alone [1] but also enables the implementation of combined electric–acoustic stimulation [2] in selected cases. Furthermore, it addresses the widespread concern of CI patients to become totally deaf.

To date, approximately 50% of CI recipients experience a loss of residual hearing within the first six months after surgery [3]. In combination with further refinement of surgical techniques (e.g., robotics-assisted electrode array insertions) and electrode array design changes, electrocochleography (ECochG) has emerged as a tool that may improve hearing preservation rates in the future. ECochG records responses to acoustic stimuli generated by hair cells as well as neural structures. However, in almost all studies concerning CI and ECochG, the cochlear microphonic (CM) as one of the hair cell components of the ECochG response has been assessed. Previous work in animals has shown that CM amplitude drops can be associated with cochlear trauma during electrode insertion [4,5,6,7,8,9]. Accordingly, multiple studies in human CI recipients have demonstrated that, on average, such amplitude drops are associated with increased loss of residual hearing after surgery [10,11,12,13,14]. However, it is also evident that not all CM drops during electrode insertion into the cochlea represent cochlear trauma. Changes of the relative contribution to the CM of different cochlear regions as the electrode is advanced into the cochlea can also lead to a decrease of the signal amplitude [15,16,17,18].

Whether individual CM drops are trauma related and relevant with regard to structure preservation or not often remains controversial. To address this issue, different approaches have been investigated. By far the most common approach is to rate amplitude drops as relevant or not, based on the extent of the amplitude drop (i.e., depending on the study relevant drops were defined as ≥3 dB, ≥6 dB or ≥30%). Drops exceeding the defined threshold are then rated as trauma related and relevant, while drops below the threshold are not [10,11,12,19]. However, this approach has two main drawbacks. First, Giardina et al. demonstrated that analyzing phase changes and distortions of the CM alongside amplitude changes improves the accuracy of predictions regarding hearing preservation [15]. Second, there is a lack of understanding of factors influencing the extent of CM drops (e.g., degree of cochlear trauma, intact cochlear structures in the region of trauma, localization of trauma). The prevailing assumption has been that the degree of cochlear trauma is the main influencing factor, but to our knowledge, experimental data confirming this hypothesis is lacking.

Since many factors potentially influencing ECochG signal changes in human CI recipients are not accessible for analysis, this study aimed to determine the influence of the degree and localization of cochlear trauma on intracochlear CM changes in normal hearing C57BL/6 mice. Normal hearing animals were chosen to have a study population as homogenous as possible, although it represents a limitation in terms of translatability to human CI recipients. Mice are a well-suited animal model for cochlear implantation due to the similarity of their auditory system to that of humans [20]. Additionally, the availability of a wide range of genetic models and consequently the future possibility to assess ECochG signals changes also in different inner ear pathologies was further incentive to choose mice as an animal model in this study. We hypothesized that the severity of cochlear trauma and, to a lesser extent, its localization could determine the magnitude of CM amplitude drops.

## 2. Materials and Methods

All animal experiments adhered to the principles of the 3Rs (Replacement, Reduction, and Refinement) and were performed according to Swiss Animal Welfare laws. All experimental protocols were approved by a named institutional committee (local veterinary authorities, permission number ZH103/2018, Kantonales Veterinäramt, Zurich, Switzerland). The results are reported in accordance with ARRIVE guidelines. Results from eleven ears in eleven female C57BL/6J-Crl1 (“C57BL/6”) wild-type mice aged 6 to 8 weeks are reported (including *n* = 2 control animals). The mice were purchased from Charles River (Freiburg im Breisgau, Germany) and housed in individually ventilated cages at an in-house animal facility. They had unlimited access to a standard chow diet and water ad libitum under a standard 12 h light/dark cycle.

### 2.1. Surgery

The mice were anaesthetized with an intraperitoneal injection of a mixture of xylazine (12 mg/kg; Rompun, Bayer HealthCare AG, Leverkusen, Germany) and ketamine (80 mg/kg; Narketan, Vétoquinol SA, 34, Magny-Vernois, France). Anesthesia depth was assessed every 15 min by checking the pedal reflex. If reflexes were present, the mice were redosed with ketamine (25 mg/kg).

**Figure 1:**
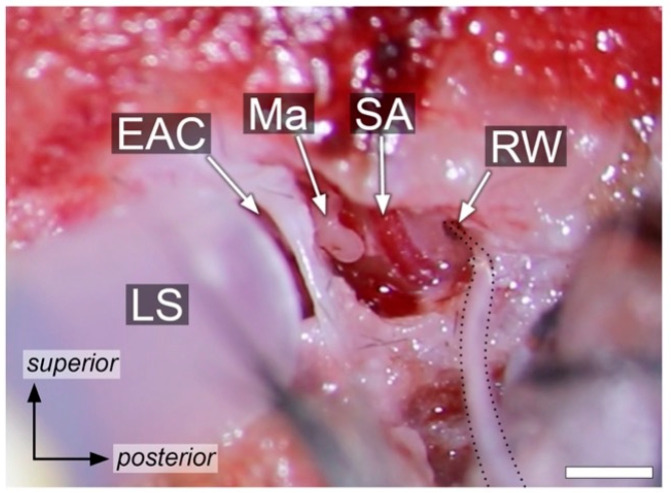
Microscope view following surgical access to the middle ear. The black dashed line outlines the electrode. EAC, external auditory canal; LS, loudspeaker; Ma, malleus; RW, round window; SA, stapedial artery. Scale bar: 1 mm.

The left retroauricular region was shaved, the mouse was placed on a heating pad, maintaining an internal temperature of 37.5 °C as monitored via rectal probe. Lidocaine (10 mg/mL; Rapidocain, Sintetica SA, Mendrisio, Switzerland) was injected in the subcutaneous tissue of the left retroauricular region for topical analgesia. Surgical access to the round window was performed using a minimally invasive technique as described previously [21] (Figure 1). Briefly, the left temporal bone was accessed via a retroauricular incision and a blunt dissection of the sternocleidomastoid muscle, the anterior scalene muscle, and the posterior belly of the digastric muscle. Bipolar electrocautery was used for dissection and hemostasis. The auditory bulla was opened using a micro drill with a 0.5 mm burr. The opening was extended until the round window was completely visualized.

The electrode (+) used for intracochlear ECochG recordings was a 25 µm diameter, Teflon-insulated platinum–iridium wire with approximately 50 µm of tip insulation removed. Consequently, the inserted electrode did not closely resemble a CI electrode array. The wire used as intracochlear electrode had a high stiffness and did not behave like a lateral wall or perimodiolar CI electrode. The reason such a stiff wire was used was that it would likely cause cochlear trauma, which was the aim of this study. Due to high stiffness, the electrode could not pass beyond the basal turn. A similar electrode to record ECochG responses and cause intracochlear trauma was used by Adunka et al. [4]. Additionally, a needle electrode was placed in the contralateral neck muscles to provide the inverting input (−), and both inputs were referenced to a needle electrode at the base of the tail (ground). An insert earphone (Biologic Systems, Mundelein, IL, USA) was positioned at the external ear canal for acoustic stimulation. The Navigator Pro stimulation/recording device and the AEP software (Version 7.0.0, Biologic Systems) were used for acoustic stimulation and recording. Tone bursts at 6 or 8 kHz with an intensity of 100 dB SPL were used as acoustic stimuli. These frequencies were chosen to resemble acoustic simulation in human CI recipients in which ECochG recordings are conducted. In human CI recipients, responses can usually only be recorded to low-frequency acoustic stimulation, and by far the most common frequency used for ECochG recordings is 0.5 kHz. In mice, the tonotopic regions of 6 and 8 kHz are comparable to the tonotopic region of 0.5 kHz in humans [22]. Recordings during insertion were conducted with only one frequency. Responses to 500 stimuli with alternating starting phases were recorded. The recorded data were exported from the AEP software using the AEP to ASCII software (Version 1.0, Biologic Systems). For post-processing, MATLAB (Version 24.1, MathWorks Inc., Natick, MA, USA) and GraphPad Prism (Version 10.4.2, GraphPad Software Inc., La Jolla, CA, USA) were used. The CM was derived by subtracting the averages of 500 responses with alternating starting phases. By subtracting both response curves with alternating starting phases, neural responses with the same polarity in both response curves are canceled out, whereas hair cell responses with different polarity in both response curves remain. A Fast Fourier Transform (FFT) was performed on the CM response to determine the peak-to-peak amplitude of each CM curve at the acoustic stimulation frequency. The amount of CM amplitude drop was defined as the difference between the last two ECochG recordings.

The round window membrane was incised, and the recording electrode was inserted in 25 µm to 50 µm steps using a manual micromanipulator (M3301, World Precision Instruments, Friedberg, Germany). At each step, the insertion was paused for the duration of the ECochG recordings. The stepwise insertion was continued up to an insertion depth where a visually detectable drop in the CM signal amplitude or resistance with further insertion occurred. In control animals, the surgical access to the round window was carried out as described above. The platinum–iridium electrode was then placed on the closed round window membrane and two ECochG recordings were conducted within a 30 min interval.

### 2.2. Histological Analysis

After completion of measurements, the animals were sacrificed during anesthesia. The platinum–iridium electrode was cut at the round window and fixed using cyanoacrylate glue. For tissue fixation, the thoracic cavity was opened, and a cannula was inserted in the left cardiac ventricle for transcardial perfusion with 10 mL phosphate buffered saline (PBS), followed by 10 mL of 10% neutral buffered formalin (catalog no. 15738-01, Lucerna-Chem AG, Luzern, Switzerland). The mice were then decapitated, and soft tissues were removed from the outer surface of the skull using a sharp scalpel. The cranial part of the skull was opened to remove the cerebrum, while the cerebellum was left in situ. The specimens were then immersed in 10% neutral buffered formalin for 24 h on a countertop shaker. After fixation, specimens were decalcified in a formic acid decalcifier for 10 days (RDF mild decalcifier, catalog no. EAA-1000-00A, CellPath Ltd., Newtown, UK). The specimens were then dehydrated in a graded series of ethanol solutions (50%, 70%, 95%, and 100%), embedded in paraffin and sectioned at 4 μm using an HM 355S Automatic Microtome (Thermo Fisher Scientific, Waltham, MA, USA). Specimens were sectioned without removing the electrodes in order to avoid removal artefacts. Sections were collected on SuperFrost Plus slides (Thermo Fisher Scientific), dried on a heating plate at 37 °C overnight and stored at room temperature. The specimens were stained with hematoxylin and eosin. Images were acquired using a Leica DMI6000 microscope (Leica, Wetzlar, Germany) and processed with Adobe Photoshop software (Version 25.0, Adobe Systems, San Jose, CA, USA). The insertion depth was measured from the round window to the tip of the electrode.

Histological trauma was rated on scale from 0 to 4 [23]: 0 represents no observable trauma; 1, elevation of the basilar membrane; 2, rupture of the basilar membrane; 3, electrode in the scala vestibuli; and 4, severe trauma such as fracture of the osseous spiral lamina or modiolus or tear of stria vascularis.

### 2.3. Micro-Computed Tomography

Micro-computed tomography (µCT; Skyscan 1176, Bruker Corp., Billerica, MA, USA) scans were conducted after completion of the above-described fixation process. The three-dimensional volumes of the cochlear structures and the inserted electrodes were reconstructed from the µCT-images using Amira software (version 6.5, Thermo Fisher Scientific Inc., Waltham, MA, USA). The insertion depth was measured from the round window to the tip of the electrode.

### 2.4. Statistical Analyses

Statistical analyses were conducted using GraphPad Prism (GraphPad Software Inc., La Jolla, CA, USA). Linear regression analyses were performed to determine the correspondence between the insertion depth of the electrode according to histological and µCT findings as well as to correlate the degree and the localization of cochlear trauma with CM amplitude changes.

## 3. Results

**Figure 2:**
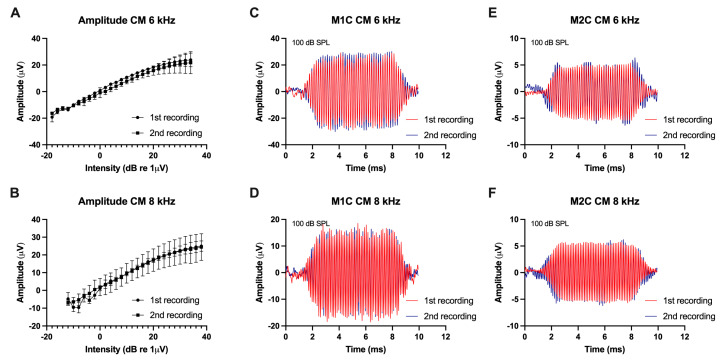
(**A**,**B**) show the mean CM curve amplitudes at different intensities at 6 and 8 kHz in control animals in both recordings (bars are standard error of the mean). (**C**–**F**) display the CM responses at 100 dB SPL at 6 and 8 kHz in both control animals.

In both control animals (M1C, M2C), no amplitude differences in the CM curve at 6 and 8 kHz could be detected between the two recordings (Figure 2).

**Figure 3:**
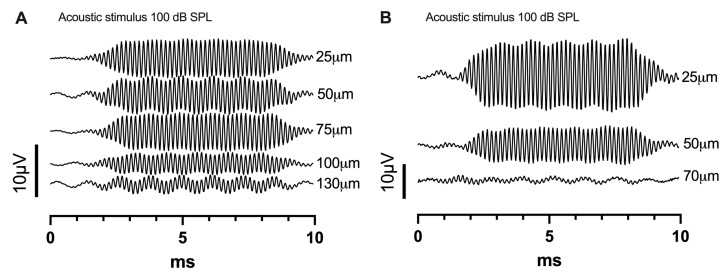
Examples of CM responses at different insertion steps are displayed. The CM amplitude drops were 9 µV ((**A**), M5) and 27 µV ((**B**), M6). These changes were associated with a histological trauma grade 2 at an insertion depth of 130 µm in M5 and grade 1 at an insertion depth of 70 µm in M6.

ECochG recordings during stepwise insertion of the electrode were performed in nine animals. The chosen frequency for acoustic stimulation was 8 kHz for *n* = 1 (M1) and 6 kHz for *n* = 8. Mean amplitude of the first CM response at the round window was 31.7 dB re 1 µV (*n* = 9, range 13.2 dB re 1 µV to 45.4 dB re 1 µV). Seven recordings showed a CM drop (mean amplitude drop 14.6 dB (*n* = 7), range 36.5 dB to 5.6 dB). Figure 3 shows two examples of CM responses at different stages of insertion. In both animals, a drop of the CM was detectable. In all animals, except M6, a CM amplitude increase was detectable during insertion of the electrode. However, it has to be noted that the amplitude increase in M5 was only minimal with 0.8 dB (Figure 3A). In M6, an amplitude drop occurred with the first insertion step; therefore, only a shallow insertion of 0.7 mm was achieved (Figure 3B). This finding was associated with very basally located cochlear trauma in the histological assessment. In two animals, the insertion was stopped due to resistance and buckling of the electrode outside of the cochlea without an associated CM drop (M7, M9). Mean insertion depth for all insertions was 1.2 mm (range 0.7 mm to 1.8 mm), which means that 10 to 29% of the cochlea were covered by the electrode and that the tonotopic region of 6 and 8 kHz, which lays between 60 to 70% of the distance from the round window, was not reached in any animal [22]. With an insertion depth of 1.2 mm, the characteristic frequency of approximately 20 kHz is reached. Histological, radiological, and electrophysiological findings are summarized in Table 1.

**Figure 4:**
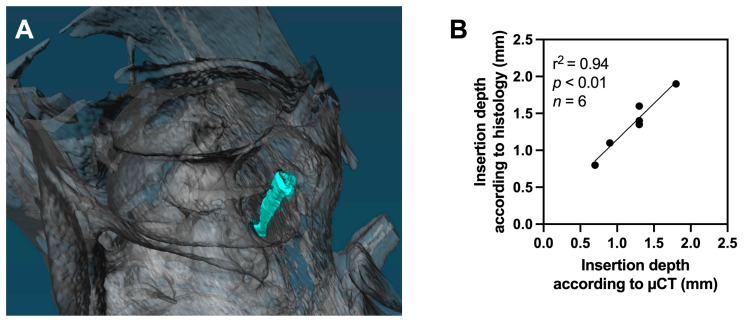
(**A**) displays an exemplary three-dimensional reconstruction of µCT-images. The electrode wire is shown in cyan blue. (**B**) shows the strong correlation between insertion depth estimation according to histological results and µCT-images.

Histology and µCT (Figure 4A) could be assessed in eight animals each. A strong correlation was observed between insertion depths estimated by histology and µCT-images when the insertion depth could be reliably assessed by both modalities (linear regression, r^2^ = 0.94, *p* < 0.01, *n* = 6, Figure 4B).

**Figure 5:**
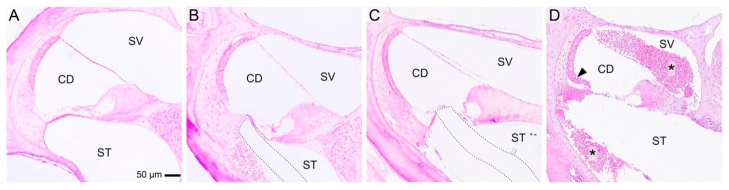
Example of microscope images of histologic trauma in the middle cochlear turn. (**A**) Normal cochlear morphology in a control animal. (**B**) Grade 1 trauma with elevation of the basilar membrane. (**C**) Grade 2 trauma with rupture of the basilar membrane. (**D**) Grade 4 trauma featuring a fracture of the osseous spiral lamina and a tear of the stria vascularis (black arrowhead). Note the extensive intracochlear bleeding (black asterisks). The dotted line outlines the electrode tract. CD, cochlear duct; ST, scala tympani; SV, scala vestibuli.

Histological trauma (grade 0–4) could be assessed in eight animals. In one animal, due to difficulties during preparation of the histological specimen, the histological sections could not be interpreted. Histological trauma was classified as grade 1 in three animals, grade 2 in four animals, and grade 4 in one animal (Table 1, Figure 5). All histological trauma was localized in the region where the tip of the stiff electrode got in contact with cochlear structures and insertion was stopped. No widespread trauma or secondary sites with cochlear trauma were detected.

**Figure 6:**
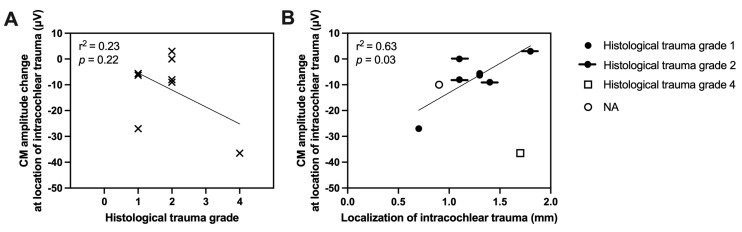
(**A**) Correlation between degree of histological trauma and the amount of CM amplitude drop. (**B**) Correlation between localization of intracochlear trauma and amount of amplitude drop. In the case of intracochlear trauma grade 1–2, the localization of the intracochlear trauma showed a significant correlation with the CM amplitude drop. Basally located intracochlear trauma was associated with larger CM amplitude drops. NA stands for not applicable.

The degree of histological trauma showed no correlation with the magnitude of the CM amplitude drop (linear regression, r^2^ = 0.23, *p* = 0.22, *n* = 8, Figure 6A). However, the localization of intracochlear trauma showed a significant correlation with the amount of CM amplitude drop in case of trauma grade 1 or 2. Basally located intracochlear trauma was associated with significantly larger CM amplitude drops (linear regression, r^2^ = 0.63, *p* = 0.03, *n* = 7, Figure 6B).

## 4. Discussion

The aim of this study was to determine factors influencing the magnitude of the CM amplitude drops observed in intracochlear ECochG recordings during electrode insertion. To correlate CM response changes with histological findings and minimize confounding factors, such as varying presurgical cochlear health, we conducted this study using normal hearing C57BL/6 mice. Our results reveal for the first time that the magnitude of the CM amplitude drop in cochlear trauma grades 1 and 2 is significantly correlated with the intracochlear location of trauma, while the degree of trauma—at least in case of grade 1 and 2 traumata—does not show such a correlation. Previous animal studies have primarily examined the relationship between cochlear trauma and the presence of an CM amplitude drop, without addressing the magnitude of these drops [4,5,6,7,8,9]. In concordance with these studies, seven out of nine animals with cochlear trauma exhibited a decrease of the CM amplitude in the study presented here. However, the correlation between localization of trauma and CM changes have never been previously explored.

The underlying mechanism for the detected correlation between trauma location and magnitude of the CM amplitude drops in cochlear trauma grade 1 and 2 remains unclear. However, defining a specific magnitude of the CM amplitude drop as a relevant indicator for cochlear trauma may be questionable, as both the occurrence and magnitude of amplitude drops are largely determined by the location of the trauma and its relation to still intact cochlear structures. Small drops may still indicate significant trauma that might not be detected by such an approach. A promising method to overcome this limitation and to gain frequency- and location-specific information about cochlear trauma could be multi-frequency and -locational ECochG recordings [16,24].

The mouse cochlea has proven to be a suitable model for the human cochlea. Structurally, the mouse cochlea is similar to the human cochlea and many genes affecting hearing in humans also cause hearing loss in mice, often with similar disease progression [25]. In this study, 6 to 8 kHz sinusoid tone bursts were used as acoustic stimuli. The tonotopic region for these frequencies in mice lays between 60 to 70% of the distance from the round window. In humans, the tonotopic region of 0.5 kHz—the frequency usually used for ECochG recordings in human CI recipients—is located in a similar location [26]. Due to the stiff wire used as a recording electrode in this study, the tonotopic region of the acoustic stimulus was not reached. Similarly, in human CI recipients, the tonotopic region of 0.5 kHz is not reached by most commercially available electrode arrays. However, it must be noted that the coverage of the cochlea by CI electrodes in humans is usually far more than in this study. In this study, only 10–29% of the mouse cochlea were covered. Consequently, the cochlear trauma described here can generally be seen as basally located trauma.

In this cohort, grade 4 cochlear trauma was observed in only one case, based on histological findings, which included a fracture of the spiral osseous lamina, disruption of the stria vascularis, and extensive intracochlear bleeding. This severe trauma was associated with an immediate and large decrease of the CM response, implying that more severe cochlear trauma with intracochlear bleeding is likely to result in substantial and immediate CM drops, even when the trauma occurs in more apical cochlear regions as in this case. However, no definitive conclusions can be drawn from this single instance. Further research with larger samples of severe trauma cases, as well as studies correlating specific trauma mechanisms (e.g., trauma to stria vascularis, to basilar membrane) with electrophysiological response patterns, is needed to advance this research field.

The results presented here suggest that grade 1 and 2 cochlear trauma can typically be detected by ECochG before more severe cochlear trauma occurs. Following the study protocol, insertion was stopped as soon as a noticeable decrease of the CM amplitude was visually observed—analogous to ECochG assessments performed in human CI recipients during surgery. This finding underscores the potential of ECochG as a valuable tool for intraoperative monitoring during cochlear implantation in the future, as it seems capable of detecting early trauma and, in some cases, potentially reversible damage, provided that intact cochlear structures remain at or apical to the trauma site.

### Limitations

Due to the design of the study with a stiff electrode inserted into the cochlea, some degree of cochlear trauma was detectable in all animals. Therefore, the research question of which CM drops represent cochlear trauma and which do not cannot be addressed. From the presented results, only some conclusions regarding factors influencing the amount of CM drops in the case of cochlear trauma can be drawn. Furthermore, due to the stiff character of the electrode, insertions beyond the basal turn were not possible. Consequently, the investigated cochlear trauma in this study only represents basal trauma. Further studies will need to investigate if ECochG changes with deeper insertions show similar characteristics.

This study was conducted in normal hearing animals, which represents a major difference compared to the highly pathological cochlea in CI recipients. Therefore, the results cannot be directly translated to human CI recipients. However, the principal mechanism that intact cochlear structures apical to the trauma site are silenced and that therefore the site of cochlea trauma and its relation to intact cochlear structures influences CM changes during insertion seems applicable to human CI recipients.

A further limitation is the absence of auditory threshold testing (e.g., auditory brainstem responses) to confirm the assumed normal hearing status of the animals before the study procedure. However, extended cochlear pathology before electrode insertion that would influence the reported study outcome seems unlikely in the chosen study animals. Additionally, in future studies, correlating ECochG changes and auditory threshold testing may allow a conclusive assessment between ECochG changes and acute hearing changes.

The fact that the insertion was stopped when an amplitude drop of the CM response was detectable and that the drop at the same time was the primary dependent variable in the analysis represents a methodological conflict. However, this approach was chosen as it reflects how ECochG responses are most often used in the operating theatre with human CI recipients. The incentive was to find out what histological and µCT findings are present in the moment the first ECochG drop occurred.

## 5. Conclusions

The location of cochlear trauma and its relation to still-intact cochlear structures have influence on the occurrence and on the magnitude of the CM amplitude drops during electrode insertion. When intact cochlear structures are present, early detection of cochlear trauma is possible. If the insertion is stopped upon detecting signal decrease, severe cochlear trauma (grade 3 or 4) seems to be rare. These findings suggest that applying this approach to cochlear implantation in human CI recipients could enhance hearing preservation during cochlear implant surgeries.

## Figures and Tables

**Table 1:** µCT stands for micro-computed tomography; NA, not applicable.

					Cochlear Microphonic Amplitude		
Animal	Histology	Histological Trauma Grade	µCT	Insertion Depth (mm)	First Recording (dB re 1 µV)	Last Recording (dB re 1 µV)	Amplitude Change (dB)
M1	Yes	4	No	1.7	37.8	12.3	−36.5
M2	No	NA	Yes	0.9	13.2	19.1	−10
M3	Yes	1	Yes	1.3	33.8	37	−6.3
M4	Yes	1	Yes	1.3	35.8	36.5	−5.6
M5	Yes	2	Yes	1.4	31	22.1	−9
M6	Yes	1	Yes	0.7	26	−1.5	−27
M7	Yes	2	Yes	1.1	30.5	34.9	0
M8	Yes	2	Yes	1.1	45.4	35.5	−8
M9	Yes	2	Yes	1.8	32.2	35.5	3

## Data Availability

The raw data supporting the conclusions of this article will be made available by the authors on request.
